# Supplementary material for: Content-rich biological network constructed by mining PubMed abstracts
Source: BMC Bioinformatics. 2004 Oct 8;5:147. doi: 10.1186/1471-2105-5-147 (PMC528731; doi:10.1186/1471-2105-5-147)
Supplement: Additional File 2 — The original results of the above study (non-essential files are deleted to keep the file size under the limit set by BMC bioinformatics). [file 1471-2105-5-147-S2.bz2 › chilibotAdditionalFile2/dip10/377ID9927421E9790/html/NGFR_NTRK1.html]

 


 **NGFR** and **NTRK1** 
  
Found 541 abstracts in PubMed, retrieved 10.  
 

 What does Google say? 
 PDF only 
| .edu only 

---

**Interactive relationship** (e.g. stimulation, inhibition, etc)

**Neutral relationship**- Although neurotrophins did not regulate p75NTR  [ **NGFR** ]  processing, the alpha and gamma secretase mediated cleavage of p75 is modulated by receptor tyrosine kinases Trks TrkA  [ **NTRK1** ]  and TrkB but not TrkC.  Ref: 12843241 J Neurosci, 2003

**Non-interactive relationship** (e.g. studied together, co-existance, homology, etc.)

- PURPOSE The purpose is to analyze the possible correlation between expression and activation of the high affinity nerve growth factor NGF receptor  [ **NGFR** ]  TrkA  [ **NTRK1** ] , cell cycle protein expression, and disease outcome in serous ovarian carcinoma.  Ref: 12796393 Clin Cancer Res< MedlineTA>Clin Cancer Res, 2003
- In the present study, the effect of signaling generated from the Erb B2 homodimer as a result of transcription of the MUC1 gene was investigated in human mammary epithelial cell lines MTSV1 7 and Hb2 stably transfected with a pBAT trk  [ **NTRK1** ]  neu construct in which the extracellular domain of Erb B2 was replaced with the corresponding domain from the nerve growth factor NGF receptor  [ **NGFR** ] .  Ref: 12798775 Cell Biol Int, 2003
- Pathways responsible for transducing the sensitisation of TRPV1 by TrkA  [ **NTRK1** ] , the NGF receptor  [ **NGFR** ] , were characterised by observing the effects of inhibitors of key members of NGF activated second messenger signalling cascades.  Ref: 12815188 J Physiol< MedlineTA>J Physiol, 2003
- Here we show that survival of sympathetic neurons supported by NGF on distal axons requires the kinase activity of the NGF receptor  [ **NGFR** ] , TrkA  [ **NTRK1** ] , in both distal axons and cell bodies.  Ref: 12848932 Neuron, 2003
- Expression and Activation of the Nerve Growth Factor Receptor  [ **NGFR** ]  TrkA  [ **NTRK1** ]  in Serous Ovarian Carcinoma.  Ref: 12796393 Clin Cancer Res< MedlineTA>Clin Cancer Res, 2003
- However, NGF rescue from growth factor withdrawal requires NGF signalling through the high affinity tyrosine kinase receptor  [ **NTRK1** ]  TrkA  [ **NTRK1** ] , while NGF rescue from TNFalpha promoted cell death requires NGF signalling through the low affinity p75NTR  [ **NGFR** ]  receptor.  Ref: 12882321 Aging Cell, 2003
- METHODS Retroviral vectors were constructed to express the high affinity NGF receptor  [ **NGFR** ]  TrkA  [ **NTRK1** ]  and low affinity NGF receptor  [ **NGFR** ]  p75 NTR.  Ref: 12877805 Chin Med J (Engl), 2003
- Quantitative in situ hybridization was used to detect changes in neuronal PACAP mRNA expression and to correlate this expression with neuronal mRNA expression of the NGF receptor  [ **NGFR** ]  tyrosine kinase trk A  [ **NTRK1** ] .  Ref: 12890505 Neuroscience, 2003
